# Navigating the dynamic landscape of long noncoding RNA and protein-coding gene annotations in GENCODE

**DOI:** 10.1186/s40246-016-0090-2

**Published:** 2016-10-28

**Authors:** Saakshi Jalali, Shrey Gandhi, Vinod Scaria

**Affiliations:** 1 GN Ramachandran Knowledge Center for Genome Informatics, CSIR Institute of Genomics and Integrative Biology (CSIR-IGIB), Mathura Road, Delhi, 110 025 India; 2 Academy of Scientific and Innovative Research (AcSIR), CSIR-IGIB South Campus, Mathura Road, Delhi, 110025 India

**Keywords:** GENCODE, Long noncoding RNAs, Transcripts, Annotations

## Abstract

**Background:**

Our understanding of the transcriptional potential of the genome and its functional consequences has undergone a significant change in the last decade. This has been largely contributed by the improvements in technology which could annotate and in many cases functionally characterize a number of novel gene loci in the human genome. Keeping pace with advancements in this dynamic environment and being able to systematically annotate a compendium of genes and transcripts is indeed a formidable task. Of the many databases which attempted to systematically annotate the genome, GENCODE has emerged as one of the largest and popular compendium for human genome annotations.

**Results:**

The analysis of various versions of GENCODE revealed that there was a constant upgradation of transcripts for both protein-coding and long noncoding RNA (lncRNAs) leading to conflicting annotations. The GENCODE version 24 accounts for 4.18 % of the human genome to be transcribed which is an increase of 1.58 % from its first version. Out of 2,51,614 transcripts annotated across GENCODE versions, only 21.7 % had consistency. We also examined GENCODE consortia categorized transcripts into 70 biotypes out of which only 17 remained stable throughout.

**Conclusions:**

In this report, we try to review the impact on the dynamicity with respect to gene annotations, specifically (lncRNA) annotations in GENCODE over the years. Our analysis suggests a significant dynamism in gene annotations, reflective of the evolution and consensus in nomenclature of genes. While a progressive change in annotations and timely release of the updates make the resource reliable in the community, the dynamicity with each release poses unique challenges to its users. Taking cues from other experiments with bio-curation, we propose potential avenues and methods to mend the gap.

**Electronic supplementary material:**

The online version of this article (doi:10.1186/s40246-016-0090-2) contains supplementary material, which is available to authorized users.

## Introduction

The last decade has seen a tremendous improvement in our ability to understand the human genome and its transcriptional output at a much higher resolution than previously possible. This has largely been possible due to the availability of technologies which have enabled the annotation of transcripts at much higher depths and resolution. A number of systematic efforts to annotate the transcriptome in the human are also worth mentioning. The earliest and most comprehensive approaches have been the H-invitational database consortium which aimed at assembling complementary DNA (cDNA) sequence information on the human genome through a global collaborative effort. This was followed by approaches including tiling arrays to characterize the transcriptional potential of the genome. Further, recent developments in deep sequencing approaches have greatly increased the resolution and facilitated the understanding of the transcriptome. Consequently, there has been the discovery of a significantly large number of novel gene loci in the genome. A large number of databases, including the ENCODE consortium, has made available gene annotations for the human genome by integrating data from the systematic explorations [1].

The efforts of the GENCODE consortium has been one of the most comprehensive and standardized approach for gene annotation and widely used by the community [1]. The initial efforts of GENCODE in the year 2008 (version 1) annotated 36,247 genes and 83,725 transcripts [2, 3] and subsequent versions of data show the annotations improve over time. The annotations were based on computational analysis, manual annotation, and experimental validation of genes and transcripts. The current release GENCODE Version 24 (V24) released in 2015 for humans has in total 60,554 genes annotated as protein-coding genes (19,815), long noncoding RNA genes (15,941), and small noncoding RNA genes (9882). It is also one of the most comprehensive annotations for long noncoding RNA genes.

Widely used by the community and constantly updated, with an average of three updates every year, we were motivated in understanding how the database evolved in the annotations, as this would provide a snapshot of the dynamic evolution of human gene annotations and specifically the long noncoding RNA annotations. We were interested in exploring both the different classes of annotations and the relative number of genes/transcripts in each annotation version towards understanding how the different gene classes and annotations evolved over time in the last decade.

We systematically analyzed the different annotations of genes/transcripts over different versions of GENCODE, starting with the first release till the latest release (V24) for the Human genome. While GENCODE serves as a major source of long noncoding RNA (lncRNA) annotations and has over time significantly and systematically catalogued the growth of lncRNA annotations, our analysis suggests a significant dynamism in gene annotations, reflective of the evolution and consensus in nomenclature of genes. We also find a number of cases where such dynamism in annotation has contributed to misannotation and in some cases results which might be highly inconsistent. An overview of the dynamism in annotation and the different facets thereof are presented.

## Results

### Data compendium of transcripts in the human genome

**Fig. 1:**
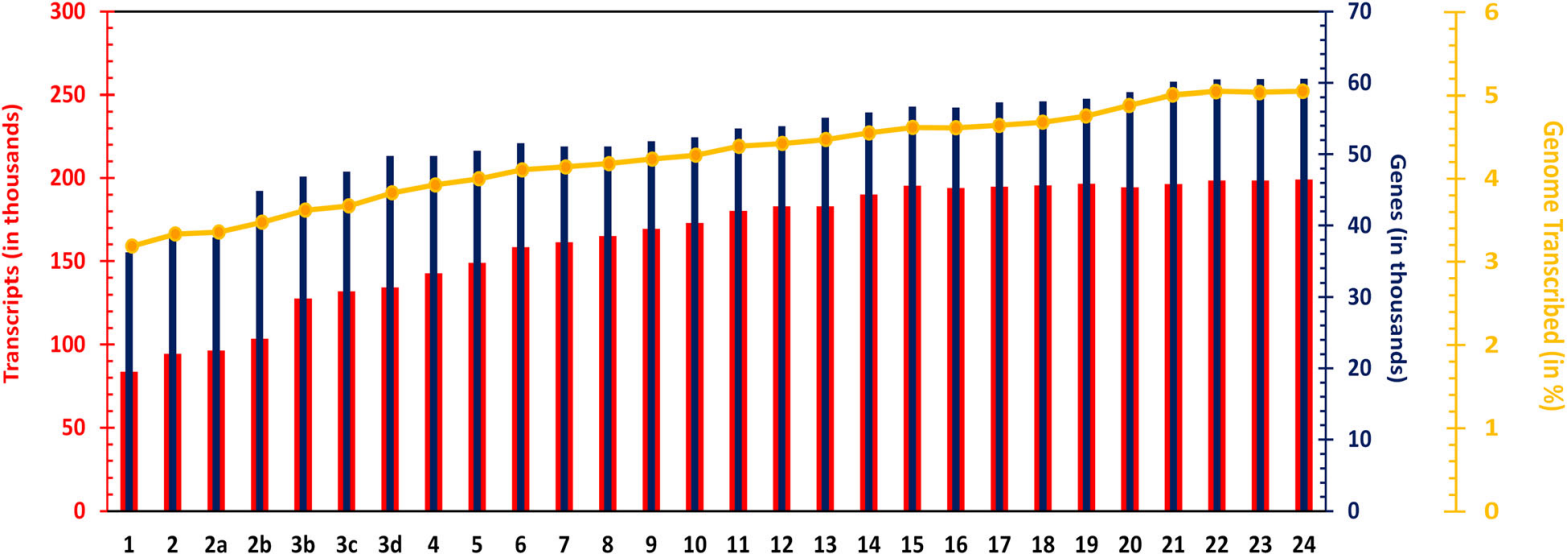
The data compendium of genes and transcripts in Human genome. The X-axis represents GENCODE versions 1-24. The Primary Y-axis (red) represents the number of transcripts (in thousand); the Secondary Y-axis (blue) represents the number of genes (in thousand) and the tertiary Y-axis (yellow) shows percentage of the genome transcribed across GENCODE versions

Through data integration of transcript information from a total of 24 versions of GENCODE from years 2008 to 2015, we assembled a large compendium of a total of 2,51,614 transcripts. The growth of GENCODE has been consistent over the different versions. The initial version started with an annotation of 87,852 transcript annotations of which 43,415 were protein-coding, while 44,437 belonged to other biotypes. The most recent version of GENCODE (V24) annotates 1,99,005 transcripts, out of which 79,865 are protein-coding while 1,19,140 belong to other RNA biotypes. The most recent annotation as per GENCODE V24 estimates approximately 4.18 % of the human genome to be transcribed, significantly up from the estimate of 2.6 % in the first version. The summary of the gene and transcript numbers, the percentage of genome transcribed as annotated in each of the versions, and their growth over the different versions is summarized in Fig. 1.

### The compendium of protein-coding and long noncoding RNA annotations

Of the entire compendium of 2,51,614 transcripts, a total of 1,14,114 transcripts were annotated as protein-coding, while a total of 1,20,864 transcripts were annotated as lncRNA biotype, in at least one of the 28 versions of GENCODE. The overlaps between these annotations revealed, a total of 11,069 transcripts had potential moonlighting identities, as shown by clashing annotations in one or the other release of the data resource. The transcripts and their overlapping annotations are summarized in Additional file 1: Figure S1.

### Growth of the compendium over time

Over years and versions, the compendium has seen significant addition of transcript annotations, with an average of 6277 additions in every new version. The largest addition to the catalog was with the V3b version in the year 2009, which saw an addition of a whopping 26,715 transcripts to the compendium. This accounted for a significant 20.91 % addition of transcript annotations to the compendium. Of these, a total of 20,499 were protein-coding transcripts, while 3096 were lncRNAs. The update also saw a deletion of 7087 transcript annotations.

While the most significant addition to the protein-coding transcript annotations occurred in V3b, the most significant addition to the lncRNA annotations happened in V4, which saw an addition of 8897 new lncRNA transcript annotations.

The consistent updates to the GENCODE compendium also saw deletion of entries in every update. On an average, 2160 transcript annotations were deleted from the database with every version. The largest deletion of transcript annotations occurred with the V20 update of the compendium in the year 2014. This update accounted for the deletion of 11,410 transcript annotations from the compendium, of which 6727 were protein-coding and 3623 were lncRNAs.

The most significant deletion of protein-coding transcript annotations occurred with V20 which saw the deletion of 6727 transcript annotations, while the most significant deletion of lncRNA annotations occurred in the V4 update which saw the deletion of 4149 transcripts. V20 was close behind with a deletion of 3623 lncRNA transcript annotations. The detail for each version is specified in Table 1.

**Table 1:** Census of transcripts and their biotypes across all GENCODE versions

S.No	GENCODE versions	Freeze year	No. of Havana transcripts	No. of Ensembl transcripts	Total transcripts	No. of Havana converted to Ensembl ID	Total number of unique transcript IDs which were considered	No. of biotypes	No. of lncRNA biotypes
1	1	2008	67,432	16,293	83,725	66,579	87,852	37	14
2	2	2009	79,899	14,505	94,404	76,890	98,855	36	14
3	2a	2009	83,049	13,352	96,401	81,833	1,01,088	35	14
4	2b	2009	83,049	20,570	10,3619	81,833	1,08,145	39	14
5	v3b	2009	7896	1,19,809	1,27,705	7669	1,27,773	38	14
6	v3c	2009	0	13,2067	1,32,067	0	1,31,891	37	14
7	v3d	2009	0	1,34,266	1,34,266	0	1,34,267	38	15
8	4	2010	0	1,42,637	1,42,637	0	1,42,467	41	15
9	5	2010	0	1,48,880	1,48,880	0	1,48,710	43	15
10	6	2010	0	1,58,489	1,58,489	0	1,58,321	44	16
11	7	2010	0	1,61,375	1,61,375	0	1,61,214	44	16
12	8	2011	0	1,65,067	1,65,067	0	1,64,906	46	18
13	9	2011	0	1,69,419	1,69,419	0	1,69,257	50	20
14	10	2011	0	1,72,975	1,72,975	0	1,72,810	51	20
15	11	2011	0	1,80,272	1,80,272	0	1,80,107	51	19
16	12	2011	0	1,83,086	1,83,086	0	1,82,921	50	19
17	13	2012	0	1,82,967	1,82,967	0	1,82,798	41	18
18	14	2012	0	1,90,051	1,90,051	0	1,89,882	41	18
19	15	2012	0	1,95,433	1,95,433	0	1,95,264	40	17
20	16	2012	0	1,94,034	1,94,034	0	1,93,865	40	17
21	17	2013	0	1,94,871	1,94,871	0	1,94,702	38	15
22	18	2013	0	1,95,584	1,95,584	0	1,95,418	38	14
23	19	2013	0	1,96,520	1,96,520	0	1,96,354	38	14
24	20	2014	0	1,94,334	1,94,334	0	1,94,173	38	14
25	21	2014	0	1,96,327	1,96,327	0	1,96,165	43	17
26	22	2014	0	1,98,442	1,98,442	0	1,98,278	47	17
27	23	2015	0	1,98,619	1,98,619	0	1,98,455	45	16
28	24	2015	0	1,99,169	1,99,169	0	1,99,005	47	18

### Consistency in annotations for protein-coding and long noncoding RNAs

**Fig. 2:**
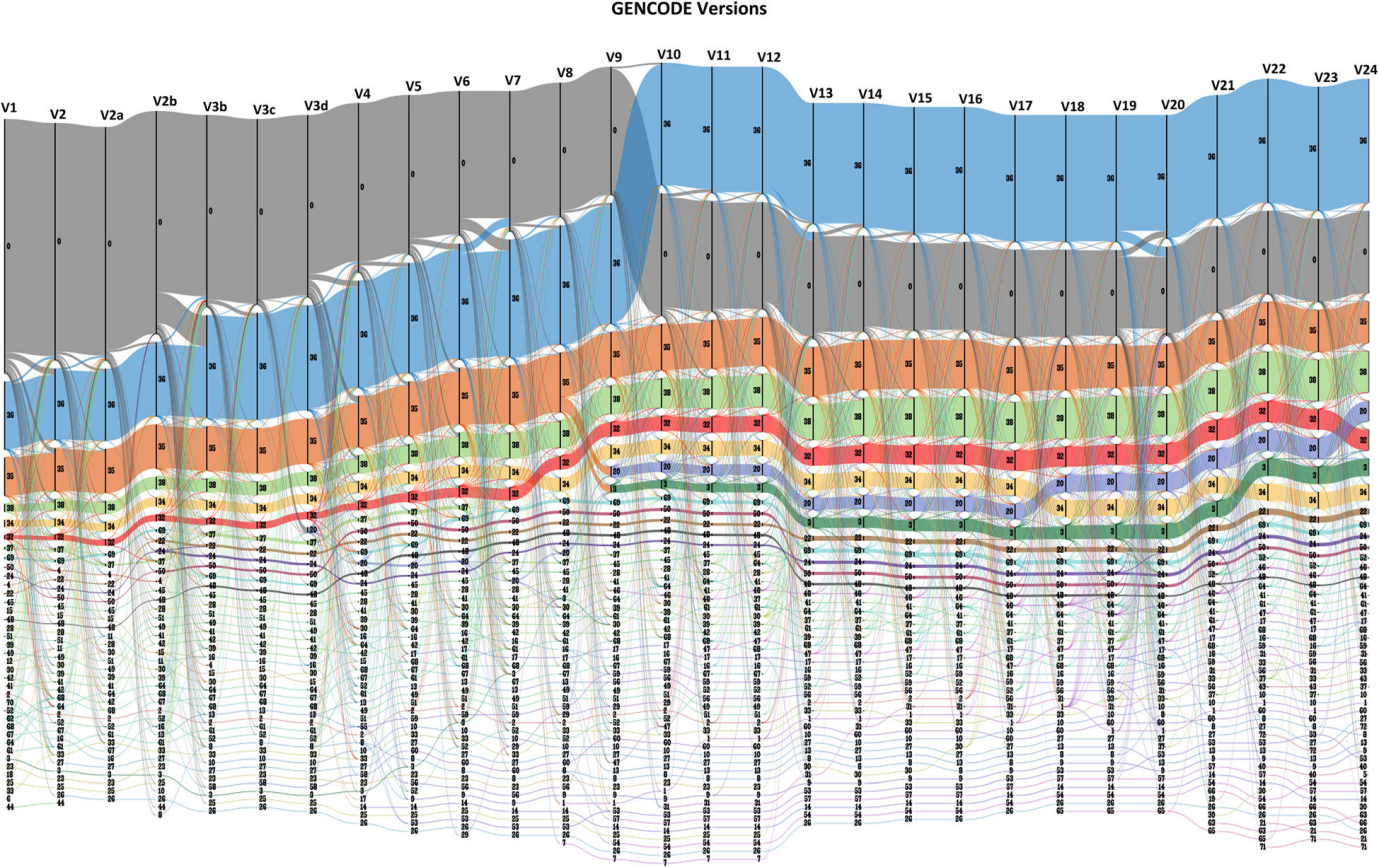
A Sankey diagram depicting the dynamicity of GENCODE biotypes across all versions (V1 to V24). The *vertical lines* represent the different versions as labeled on the top. The *horizontal lines* represent individual transcripts having any of the 72 biotypes. The biotype has been labeled as numbers as detailed in Table 4. The NA class of transcripts defined here represents the number of transcripts which were deleted in each of the versions or the number of transcripts which do not exist in each version and have represented with zero (0). The thickness of *horizontal lines* represents the number of transcripts having that particular biotype in individual versions

Of the total number of transcripts, a total of 54,840 consistently maintained their annotations across all the GENCODE versions. Of these, 32,458 were protein-coding transcripts, while 22,382 belonged to other RNA biotypes. Out of the consistent transcript annotations throughout the versions, 19,520 belonged to lncRNAs. The dynamicity of the GENCODE compendium is summarized in Fig. 2.

### Dynamicity of the lncRNA compendium and transformation of annotations

Out of this compendium, a total of 1,37,909 were annotated as noncoding RNA in one of the versions of GENCODE, of which a significant number amounting to 29,512 transcripts were systematically and consistently annotated as lncRNAs in all of the 24 versions. This accounted for 24.41 % of the total lncRNA annotations.

Of the total of 10,718 transcripts which had fleeting identities, a significant number of annotations were from a protein-coding biotype to lncRNA, which accounted to 6560 transcripts, while the reverse accounted for 5463 transcripts in total. A total of 650 lncRNA transcript annotations reversed back after moonlighting as a protein-coding transcript, while 688 protein-coding transcripts reverted back after moonlighting as an lncRNA.

This dynamic nature of transcript biotypes was consistently observed across all the updates to the GENCODE compendium. The most significant change in the protein-coding transcript annotations happened in V3b leading to 20,499 transformations. In V4, had the most significant change in the lncRNA annotations wherein 10,044 transcripts changed their annotations to lncRNA while simultaneously 4498 lncRNA transcripts mutated their annotations to other biotypes. The largest change from the protein-coding transcripts to other biotypes occurred with V20 update of the compendium in 2014 which accounted for 7212 transcripts. The detail for each version is specified in Table 2.

**Table 2:** Details of all the biotypes used in GENCODE and their respective codes as used in our study

Biotype name	Code given
3 prime overlapping ncrna	1
Ambiguous orf	2
Antisense	3
Artifact	4
Bidirectional promoter lncrna	5
C segment	6
Disrupted domain	7
IG C gene	8
IG C pseudogene	9
IG D gene	10
ig gene	11
IG gene	12
IG J gene	13
IG J pseudogene	14
IG pseudogene|ig pseudogene	15
IG V gene	16
IG V pseudogene	17
J segment	18
Known ncrna	19
lincRNA	20
macro lncRNA	21
miRNA	22
miRNA pseudogene	23
misc RNA	24
misc RNA pseudogene	25
Mt rRNA	26
Mt tRNA	27
Mt tRNA pseudogene	28
ncrna host	29
Non-coding	30
Non-stop decay	31
Nonsense-mediated decay	32
Polymorphic pseudogene	33
Processed pseudogene	34
Processed transcript	35
Protein coding	36
Pseudogene	37
Retained intron	38
Retrotransposed	39
Ribozyme	40
rRNA	41
rRNA pseudogene	42
scaRNA	43
scRNA	44
scRNA pseudogene	45
Sense intronic	46
Sense overlapping	47
snoRNA	48
snoRNA pseudogene	49
snRNA	50
snRNA pseudogene	51
TEC|tec	52
TR C gene	53
TR D gene	54
TR gene	55
TR J gene	56
TR J pseudogene	57
TR pseudogene	58
TR V gene	59
TR V pseudogene	60
Transcribed processed pseudogene	61
Transcribed pseudogene	62
Transcribed unitary pseudogene	63
Transcribed unprocessed pseudogene	64
Translated processed pseudogene	65
Translated unprocessed pseudogene	66
tRNA pseudogene	67
Unitary pseudogene	68
Unprocessed pseudogene	69
V segment	70
Vaultrna	71
sRNA	72

### Differences in the biotypes and annotations between versions of GENCODE

**Fig. 3:**
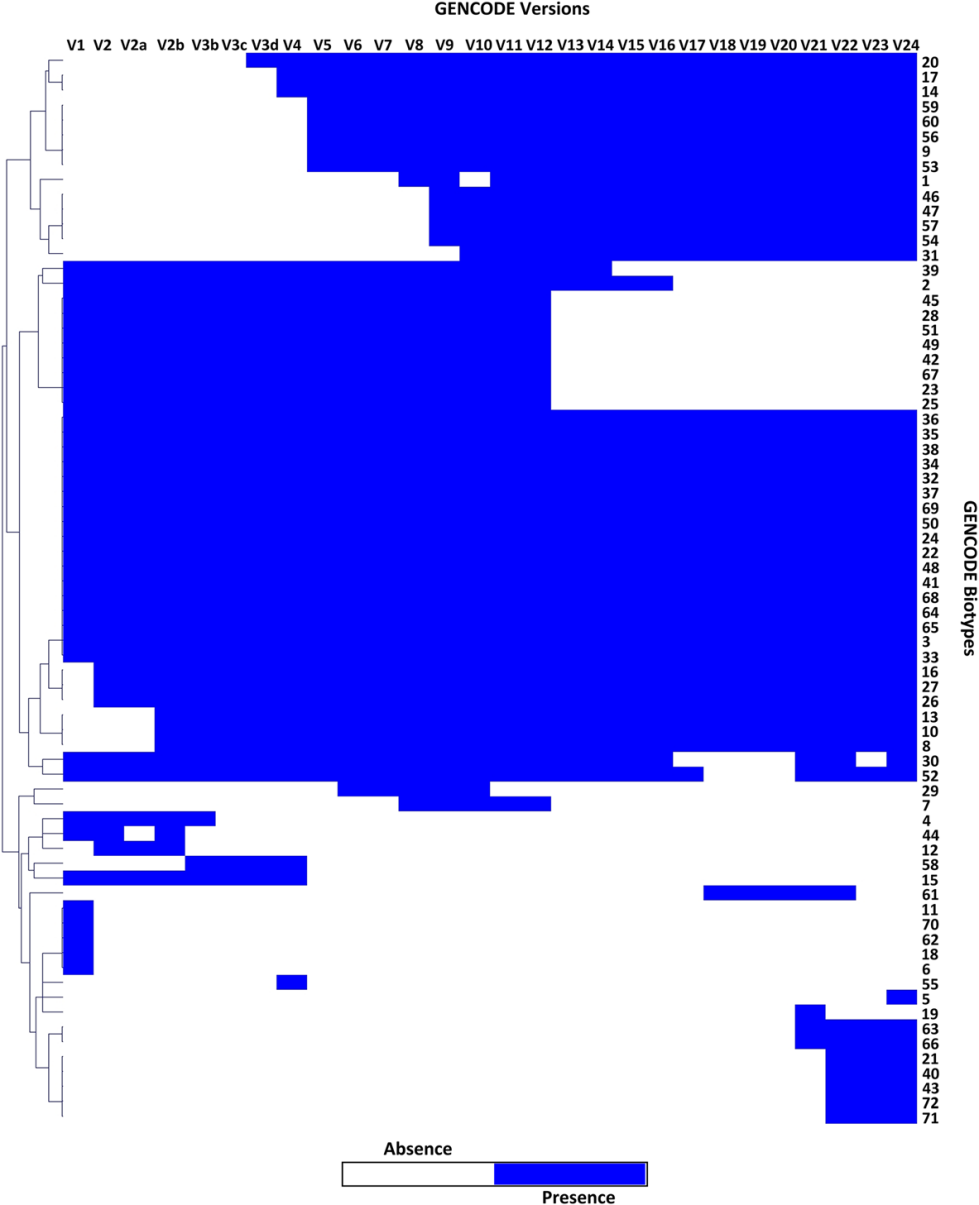
Heatmap depicting the presence and absence of each biotype across different GENCODE versions. The *blue color* represents presence of a biotype, and the *white color* represents absence of a biotype. The *Y*-axis lists all the 71 biotypes and X-axis has all the GENCODE versions

We evaluated the dynamicity in the biotypes under which the transcripts were annotated in different versions of GENCODE. Our analysis revealed a total of 70 biotypes were considered in total for annotation of transcripts. Only a small proportion (17) of their entire compendium of biotypes was systematically used in all the versions of GENCODE. A subset of 9 (Ambiguous ORF, scRNA pseudogene, Mt tRNA pseudogene, snRNA pseudogene, snoRNA pseudogene, rRNA pseudogene, miRNA pseudogene, misc RNA pseudogene) biotypes were dropped after v12, while 12 (ncRNA host, Disrupted domain, TR pseudogene, Artifact, scRNA, TR gene, IG gene, V segment, transcribed pseudogene, J segment, C segment) biotypes were used only in the earlier versions of GENCODE. The presence and absence of all biotypes across various versions of GENCODE are summarized in Fig. 3.

### Impact of dynamicity of the lncRNA compendium

We also evaluated the impact of the dynamicity of annotations. Our analysis revealed a total of 1,96,988 transcripts had a dynamic annotation in at least one of the versions of GENCODE. This accounted for a total of 78.29 % of all the transcript annotations in GENCODE.

We closely examined a few candidates which had a significant dynamicity in its annotation (as shown in Additional file 2: Figure S2). We selected candidates which over versions of GENCODE have been dynamically annotated as a protein-coding or long noncoding RNA. One such candidate is C3orf10 (ENST00000256463). C3orf10 gene encodes for a 9-kD protein which plays a role in regulation of actin and microtubule organization. This gene encodes for ENST00000256463 which was annotated as protein coding in V1 then as an lncRNA in V2-V2a and V3c-V6 and later again annotated as protein coding and further dropped from the database since version 20. In addition to inconsistency to the annotation type, it also had different gene names across versions the name of this transcript also changed: C3orf10 (V1-V8) -> AC034193.5 (V2-V3b) -> BRK1 (V9-V19). There were also few transcripts which had consistently same name such as ENST00000436930: FER1L5 (V1-V24), ENST00000366438: ATAD2B (V1-V24) across the entire version with varying annotations. While few transcripts such as ENST00000334998: RP1-163 M9.4 ( V1-V2b) -> MST1P9 (V3b-V14 ) -> MST1L (V15) -> current status does not exist, ENST00000339140: RP11-167P23.5 (V1-2b) -> FOXR2 (2b-V24), ENST00000408914: RIMKLP (V1-V3d) -> RIMKLB2 (V4-V5) -> RIMKLBP1 (V6-V24) and had both inconsistent name as well as biotype.

Another example from our analysis is AC074389.6 gene which encodes for a single transcript (ENST00000382528) according to GENCODE annotations. It was annotated as protein coding in V1- 20 and this transcript is annotated as lincRNA from V21. This gene was identified as a novel bioactive peptide in year 2006 derived from precursor proteins which can be used as targets for drug interventions. To identify this new gene, the human genome National Center for Biotechnology Information (NCBI) 33 assembly, July 1, 2003, was used as reference and novelty of peptide sequence was confirmed using Universal Protein Resource (UNIPROT) [4]. Expression profile studies were also conducted to show their presence in various tissues [5]. Recently, Wang et al. reported this transcript to be expressed as an Lnc-RI lncRNA, and the same was shown through experimental validation to be ubiquitously expressed [6]. These contrasting reports highlight the genuine concern which arises due to frequent and ever changing landscape of GENCODE annotations.

The transcript ENST00000413529, encoded by the gene SDHAP3, was the most inconsistent transcript across the entire GENCODE compendium, which witnessed a total of nine transitions and was assigned six different biotypes during its short lived journey (V3b-19) Additional file 3: Figure S3.

Using HGNC (The HUGO Gene Nomenclature Committee) [7], one of the largest consortium of the human genes, we wanted to check the existence of the deleted genes in the present GENCODE(V24). The total human gene list extracted from HGNC consisted of 39,777 loci, and there were total of 56,095 GENCODE genes which were present in the earlier GENCODE versions but got eliminated in the current version (V24). When we overlapped the current HGNC genes with the genes deleted in V24, we found 285 genes to be common, out of which, 35 were lncRNAs. The same is depicted in Additional file 4: Figure S4.

## Discussion

The GENCODE compendium of transcript annotations has undoubtedly significantly enhanced the accessibility to a standardized set of genome annotations and accelerated the experimental annotation and understanding of gene functions, especially long noncoding RNA functions. Though there have been a number of databases [8] systematically annotating various aspects of lncRNAs including their functions, interactions etc., all the databases have been lacking continuous updates. GENCODE fills in this gap by covering and integrating the latest in terms of gene and transcript annotation, methodologies, and standards. Notwithstanding the limitations of the resource, which primarily arise from the changing landscape of technologies, definitions and methods for transcriptome analysis, GENCODE still provides one of the most comprehensive and well-accepted compendium of transcript annotations widely used and followed in literature.

A major limitation of the field has been the inconsistency in the nomenclature of transcript/gene biotypes which significantly adds confusion in the classification and long-term annotation of transcripts, especially lncRNAs. Our analysis of GENCODE suggests that a significant number of 52 biotype annotations were dropped at one point or the other between different versions of GENCODE, which affects a total of 1,96,799 transcript annotations while 17 biotypes remained constant across all GENCODE version for 54,815 transcripts.

In a very dynamic technological and knowledge landscape, it would be imperative for resources to closely integrate the long tail of annotations. It is humanly impossible for organizations to systematically track the growing corpus of literature in the field (Additional file 5: Figure S5), which presently adds over 1000 new publications per year. Therefore, it is imperative to dynamically interlink publications and resources related to the field as has been extensively built for protein-coding genes [7].

Another major gap in the field has been the lack of interoperable databases annotating different biological aspects of lncRNAs. Apart from the standard Ensembl IDs followed by GENCODE and used by many other databases, only a small proportion of the lncRNAs 1.46 % of the entire compendium of lncRNAs have also been annotated and provided an HGNC gene symbol. Apart from the standard HGNC gene nomenclature, many publications and resources cite a variety of other nomenclatures, which adds to the confusion and inability to cross-link resources, publications, and analysis results. This major limitation stems from that fact that there has been a lack of standard and consensus standards for nomenclature of lncRNAs. Such standards for nomenclature and annotation of many other noncoding classes including miRNAs have ensured accordance in nomenclature which in turn maintains the compatibility between resources, databases, and citations in publications [7, 9, 10].

A number of resources and databases on lncRNAs have emerged in the recent years and has been comprehensively reviewed by Jalali and co-workers [11]. The resources encompass a variety of biological relationships, interactions, and functionalities. Nevertheless, the integration of the resources into a common platform has been a tedious task due to the variability in annotation standards, version of the annotations used, and lack of interoperability between the resources. The immediate goal would be to enable these complementary resources to be interoperable. The availability of common standards for nomenclature and annotation would enable the resources to be systematically integrated which would in turn enable timely updates. This would facilitate experimental as well as computational biologists wade through the unchartered waters quickly, and effectively.

The update in this ever-growing field has been fast outpacing the efforts by individual groups or laboratories to be able to systematically curate the information in a comprehensive way. Different attempts to fill in the gap of the long tail of bio-curation has emerged in the recent years, including Wiki-based systems for systematic and real-time annotation and curation of biological information. Such resources have been extensively developed not just for model systems but also for noncoding RNA databases. This could be complemented by efforts to automatically tag and annotate data from publications and resources using machine learning approaches developed recently [12].

## Conclusion

In summary, our analysis of one of the most comprehensive resource of lncRNAs suggest the dynamic progression of the field in terms of both the number of annotations as well as the changing view of the classification of lncRNAs. While a dynamic change in annotations and a timely release of the updates make the resource unique, popular, and therefore widely used by the community, the dynamicity poses unique challenges to the community. Taking cues from other domains of bio-curation, we propose modalities to mend the gap.

## Methods

### GENCODE annotation

We downloaded the annotation data in form of Gene Transfer File (GTF) files from the GENCODE database and extracted all the transcript IDs along with their corresponding biotypes across all the versions from V1 to V24. GENCODE consortium has not made available Version 3a publically, hence not included in our study. The census for transcripts and biotypes across versions is detailed in Table 1. There are 28 GENCODE releases in our analyses consisting of genomic elements such as genes, transcripts, Coding sequence (CDS), untranslated regions (UTRS), and Exons annotated by Ensembl and Havana (Human and Vertebrate Analysis and Annotation). These were classified into 71 different biotypes as listed in Table 2 across all versions.

### Analysis of consistency of transcripts across GENCODE versions

We extracted all the transcript identifiers comprising of both ENST (Ensembl) and\or OTTHUMT(Havana) IDs along with their transcript type. V1 consisted of only annotations for exons with no separate records for the other genomic elements such as genes, transcripts, or CDS. Hence, we directly used the transcript IDs as assigned to these exons for further analysis.

GENCODE assigned ENSTR/ENSTRR identifiers for pseudo autosomal regions of Y chromosome which are same for the X and Y chromosomes. For our analysis, we replaced all such transcripts with their respective ENST0 IDs in order avoid duplicate entries. We replaced 218 ENST0 IDs with their respective ENSTR /ENSTRR IDs if they had the same ENST identifier and biotype in a particular version.

Moreover, the earlier versions (V1 to 2c) of GENCODE consisted of either OTTHUMT or ENST identifiers for all transcripts. From V3b, GENCODE started to assign both the identifiers to most of the transcripts with an exception of a few which were assigned only IDs prefixed with OTTHUMT. After V3c the OTTHUMT prefixed IDs were systematically phased out as the main identifier, with each transcript having an ENST prefixed ID along with its corresponding OTTHUMT prefixed identifier. 77,193 OTTHUMT prefixed IDs had single ENST prefixed ID throughout their lifetime and hence were replaced with their respective ENST prefixed IDs. While 1982 OTTHUMT prefixed IDs had more than one ENST IDs in the same version therefore such OTTHUMT prefixed IDs were duplicated by assigning them both the Ensembl prefixed IDs while keeping their biotypes intact.

Another set of 3188 OTTHUMT prefixed IDs having more than one ENST prefixed IDs assigned to them across versions were replaced with respective IDs in that version by keeping the biotype of OTTHUMT prefixed ID intact. In addition, for 3272 OTTHUMT prefixed IDs there existed no ENST prefixed ID hence we kept them as it is.

All these transcripts IDs along with their assigned biotypes were organized into compiled record of total annotations. Those transcripts which did not have any biotype assigned to them in GENCODE versions were given a hypothetical code NA (not assigned). All the computation was performed by using custom shell and Perl scripts.

### Analysis of consistency of lncRNA transcripts across GENCODE versions

To analyze the distribution and dynamism of lncRNA annotations across the GENCODE versions, we compared the lncRNA biotypes assigned by GENCODE. We made a comprehensive list of all the lncRNA biotypes or transcript biotypes used and dropped across the different versions (as listed in Table 3). While considering lncRNA as a class, we clubbed 23 sub-biotypes, namely 3 prime overlapping ncrna, TEC, Ambiguous orf, Antisense, Bidirectional promoter lncrna, Disrupted domain, Known ncrna, lincRNA, macro lncRNA, misc RNA, ncrna host, Non coding, Processed pseudogene, Processed transcript, Pseudogene, Retained intron, Retrotransposed, Sense intronic, Sense overlapping, Transcribed processed pseudogene, Transcribed unprocessed pseudogene, Unitary pseudogene, and Unprocessed pseudogene. From the compiled record of complete annotations, we extracted the transcripts belonging to these lncRNA subclasses and named it as lncRNA annotations.

**Table 3:** Number of transcripts added or deleted in each version of GENCODE

GENCODE version	Transcripts added	lncRNAs added	PC transcripts added	Transcripts deleted	lncRNAs deleted	PC transcripts deleted
1	–	–	–	–	–	–
2	13,568	7455	4156	2565	357	1690
2a	5580	3195	1769	3347	1756	1326
2b	7069	1243	1606	12	7	0
v3b	26,715	2924	19,998	7087	1666	1674
v3c	4978	1606	2643	860	169	143
v3d	3581	3481	96	1206	192	967
4	15,138	8897	3786	6937	4149	1481
5	7065	3820	2443	822	323	221
6	10,409	5527	3838	798	156	616
7	11,285	3234	7524	8392	2519	5834
8	5036	2750	1784	1344	61	1268
9	4568	2551	1582	217	67	146
10	3684	2171	1169	131	28	102
11	7817	4801	2290	520	463	56
12	3243	1808	1096	429	237	155
13	6734	3393	1391	6857	120	5272
14	7291	4013	2543	207	118	77
15	5749	3237	2079	367	214	107
16	628	451	132	2027	1052	812
17	1469	1194	206	632	340	185
18	1055	778	158	339	204	109
19	1378	1147	192	442	234	176
20	9229	3676	4238	11,410	3623	6727
21	2218	1709	432	226	119	97
22	2873	1630	751	760	268	320
23	350	212	104	173	117	52
24	758	473	206	208	71	105

### Visualization

**Fig. 4:**
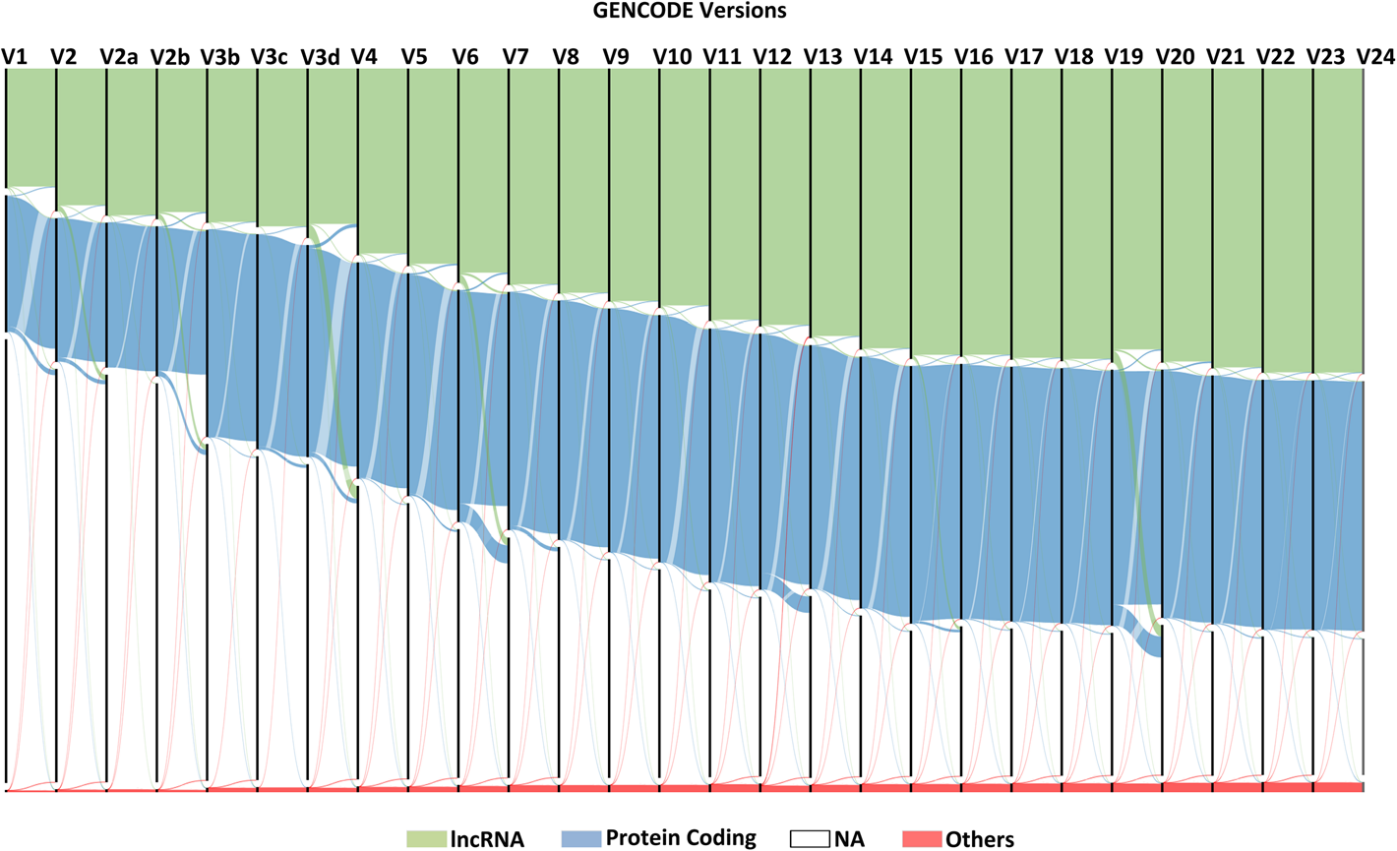
A Sankey diagram depicting the dynamicity of GENCODE lncRNAs and protein-coding biotypes across all versions (V1 to V24). The lncRNA class considered here covers 23 sub-biotypes which includes 3 prime overlapping ncrna, TEC, Ambiguous orf, Antisense, Bidirectional promoter lncrna, Disrupted domain, Known ncrna, lincRNA, macro lncRNA, misc RNA, ncrna host, Non coding, Processed pseudogene, Processed transcript, Pseudogene, Retained intron, Retrotransposed, Sense intronic, Sense overlapping, Transcribed processed pseudogene, Transcribed unprocessed pseudogene, Unitary pseudogene, Unprocessed pseudogene. The protein-coding class represents the number of transcripts having protein-coding biotype. The NA class of transcripts defined here represents the number of transcripts which were deleted in each of the versions or the number of transcripts which do not exist in each version. While the others category comprises rest of biotypes

The distribution of all the transcripts in conjunction with their biotypes across the GENCODE versions from the compiled record for total annotations was visualized using an open web app, RAW [13]. A custom vector-based visualization based D3.js library through an interactable interface was used. The dynamicity of GENCODE annotations across all versions was depicted in form of a Sankey diagram (Fig. 2). In addition, we plotted a Sankey using lncRNA annotations file, as depicted in Fig. 4. Here, we considered four categories, namely lncRNA, protein coding, NA, and others (which included all other biotypes).

We also explored the disparity of biotypes across the GENCODE annotations. Hence, we considered the all the biotypes across different versions and plotted them in form of a heatmap. We observed many biotypes which were eliminated completely while few were retained throughout (Fig. 3).

### Comparison across GENCODE versions

We calculated the number of transitions which each transcript went through during their lifetime which has been outlined in the Table 4. We also computed the various biotypes which each transcript was assigned and compiled this information in Table 5.

**Table 4:** Summary of the number of biotypes assigned to each of the transcripts

No. of biotypes assigned to the transcript	No. of transcripts
1	54,840
2	1,74,779
3	20,528
4	1945
5	256
6	41
7	5

**Table 5:** Summary of the number of transitions each transcript went through

No. of transitions	No. of transcripts
0	54,840
1	1,33,630
2	55,951
3	6420
4	1125
5	283
6	95
7	35
8	12
9	3

A compilation of the number of transcripts which were added and deleted in each version of GENCODE was derived from the compiled record of complete annotations. We also did this for both lncRNA and protein-coding transcripts which has been added/deleted, and the same has been outlined in the Table 1.

While the above table depicted the number of added/deleted transcripts, we also wanted to highlight the different transitions which these protein-coding and lncRNA transcripts went through across the GENCODE versions. Thus, on similar lines, we also produced a table outlining the switching of these transcripts which has been demonstrated in the Table 6.

**Table 6:** Switching of transcripts across versions

GENCODE version	Transcripts added	Transformed to lncRNAs	Transformed to PC transcripts	Transcripts deleted	Transformed from lncRNAs transcripts	Transformed from PC transcripts
1	–	–	–	–	–	–
2	13,568	7781	4336	2565	580	2000
2a	5580	3296	5354	3347	1834	96
2b	7069	1261	1687	12	7	0
v3b	26,715	3096	20,499	7087	2255	2049
v3c	4978	1611	2665	860	194	162
v3d	3581	3722	96	1206	189	1210
4	15,138	10,044	4073	6937	4498	2717
5	7065	4078	2662	822	593	521
6	10,409	6141	4261	798	714	1266
7	11,285	3874	8325	8392	3292	6519
8	5036	2933	1868	1344	155	1508
9	4568	2762	1677	217	178	282
10	3684	2284	1257	131	119	235
11	7817	5028	2530	520	855	322
12	3243	2069	1273	429	469	428
13	6734	4244	1679	6857	557	5660
14	7291	4314	2927	207	631	415
15	5749	3364	2201	367	384	278
16	628	649	311	2027	1271	1019
17	1469	1480	415	632	677	474
18	1055	940	385	339	481	275
19	1378	1289	474	442	582	334
20	9229	4125	4861	11,410	4324	7212
21	2218	2263	535	226	231	618
22	2873	1820	838	760	390	503
23	350	277	195	173	231	112
24	758	527	300	208	197	165

We also analyzed the abundance of publications for long non coding RNAs over last decade, for which we derived the year wise publication list from Pubmed by searching keyword “lncRNA.” The graph shown in Additional file 3: Figure S3 gives a brief layout of the number of publication per year.

### Comparison with HGNC

HGNC is the largest and one of the most reliable sources for which assigns unique and standardized nomenclature for human genes created as part of the Human Genome Organization (HUGO) [7]. We wanted to verify whether the genes which do not exist in the present GENCODE version are still present in HGNC. Thus, we extracted all the HGNC genes having approved HGNC IDs (up till last updated: 05/07/16 04:51:01) and checked their presence in last V24.

### Data availability

The detailed methodology along with all the associated content used in our analysis is available as a GitHub repository (https://github.com/vinodscaria/Gencode-moonlighting/blob/master/README.md). All other relevant data are within the paper and its supporting information.

## Additional files

Additional file 1:
**Figure S1.** Venn diagram representing the moonlighting of lncRNA and protein-coding transcript annotations. (JPG 1090 kb)
Additional file 2:
**Figure S2.** Heatmap depicting transitions of the six candidate transcripts from Protein-coding biotype to lncRNA biotype or vice versa over the different versions of GENCODE. (JPG 1418 kb)
Additional file 3:
**Figure S3.** The transition of ENST00000413529 (SDHAP3) transcript over the various GENCODE versions. (JPG 606 kb)
Additional file 4:
**Figure S4.** Common and unique annotated genes of absent in GENCODE V24 and HGNC. Venn diagram shows intersection between genes annotated by GENCODE and HGNC. (JPG 1073 kb)
Additional file 5:
**Figure S5.** Growth of literature in the field of lncRNAs. The number of publications for each year was retrieved using keyword “lncRNA” from PubMed. The data for 2016 is incomplete at the time of writing the manuscript and therefore marked with dotted lines. (JPG 1837 kb)

## Abbreviations

cDNA: Complementary DNA
CDS: Coding sequence
ENST: Ensembl identifier
GENCODE(V1-24): GENCODE(Version1-24)
Havana: Human and Vertebrate Analysis and Annotation
HGNC: The HUGO Gene Nomenclature Committee
HUGO: Human Genome Organization
lncRNA: Long noncoding RNA
NCBI: National Center for Biotechnology Information
UNIPROT: Universal Protein Resource
UTR: Untranslated region
