# Supplementary figures and images for: Navigating the dynamic landscape of long noncoding RNA and protein-coding gene annotations in GENCODE
Source: Hum Genomics. 2016 Oct 28;10:35. doi: 10.1186/s40246-016-0090-2 (PMC5084464; doi:10.1186/s40246-016-0090-2)

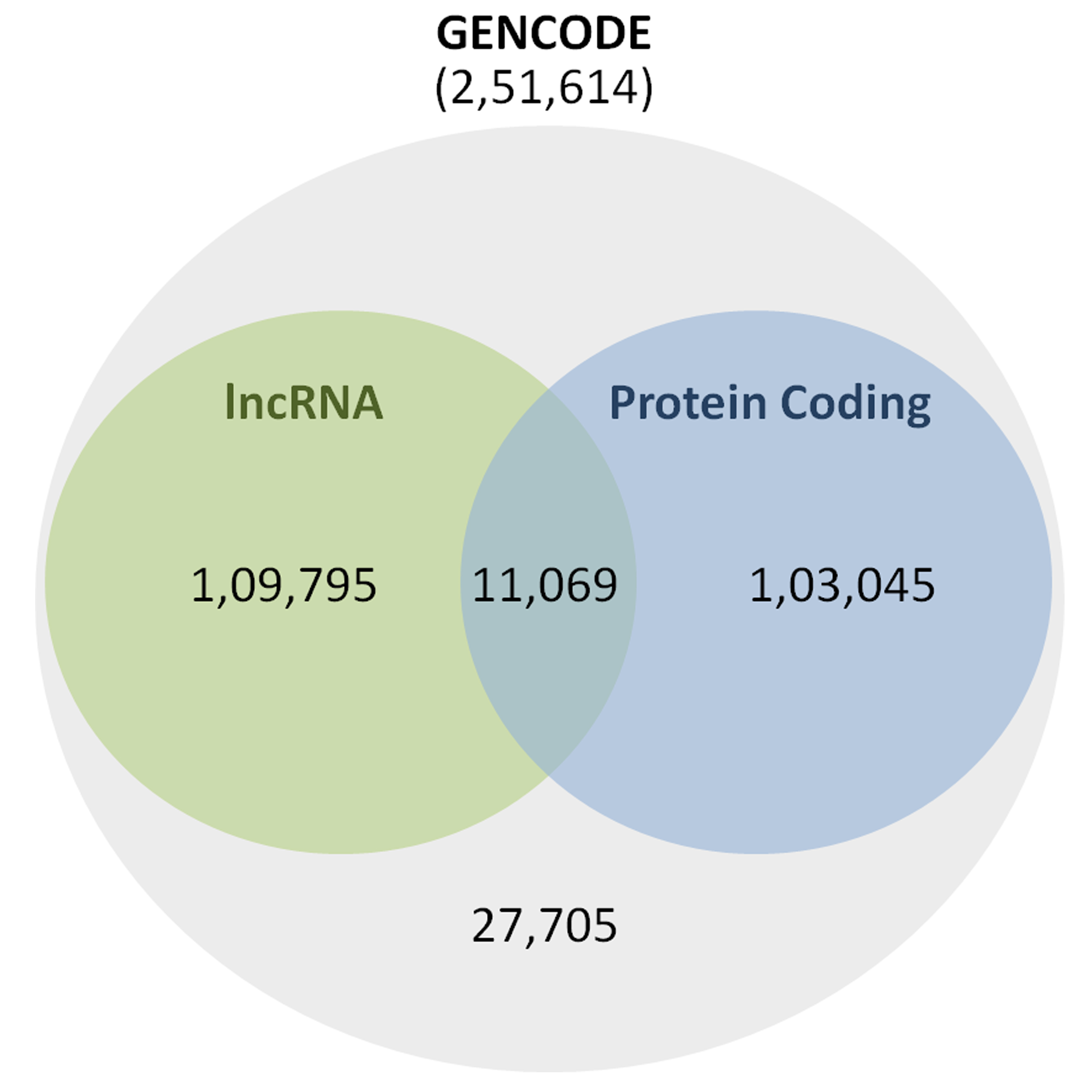

Supplement: Additional file 1: — Figure S1. Venn diagram representing the moonlighting of lncRNA and protein-coding transcript annotations. (JPG 1090 kb) [file 40246_2016_90_MOESM1_ESM.jpg]

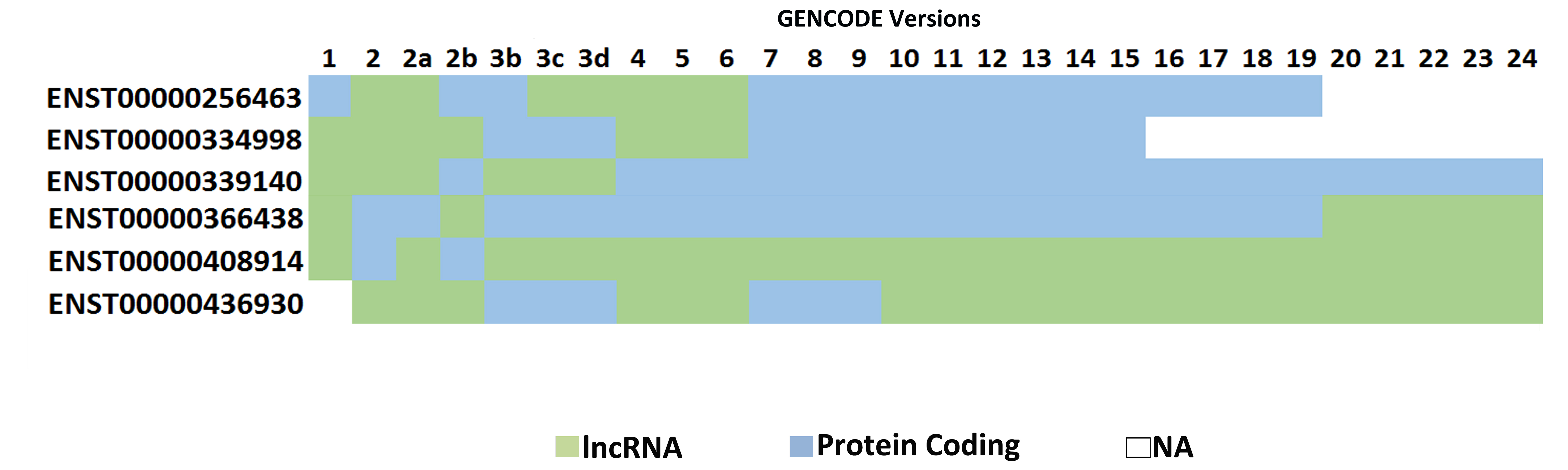

Supplement: Additional file 2: — Figure S2. Heatmap depicting transitions of the six candidate transcripts from Protein-coding biotype to lncRNA biotype or vice versa over the different versions of GENCODE. (JPG 1418 kb) [file 40246_2016_90_MOESM2_ESM.jpg]

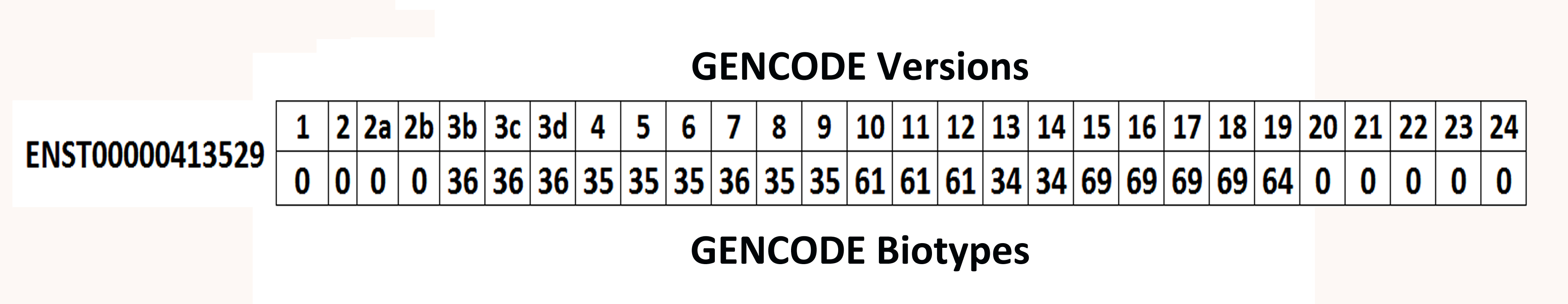

Supplement: Additional file 3: — Figure S3. The transition of ENST00000413529 (SDHAP3) transcript over the various GENCODE versions. (JPG 606 kb) [file 40246_2016_90_MOESM3_ESM.jpg]

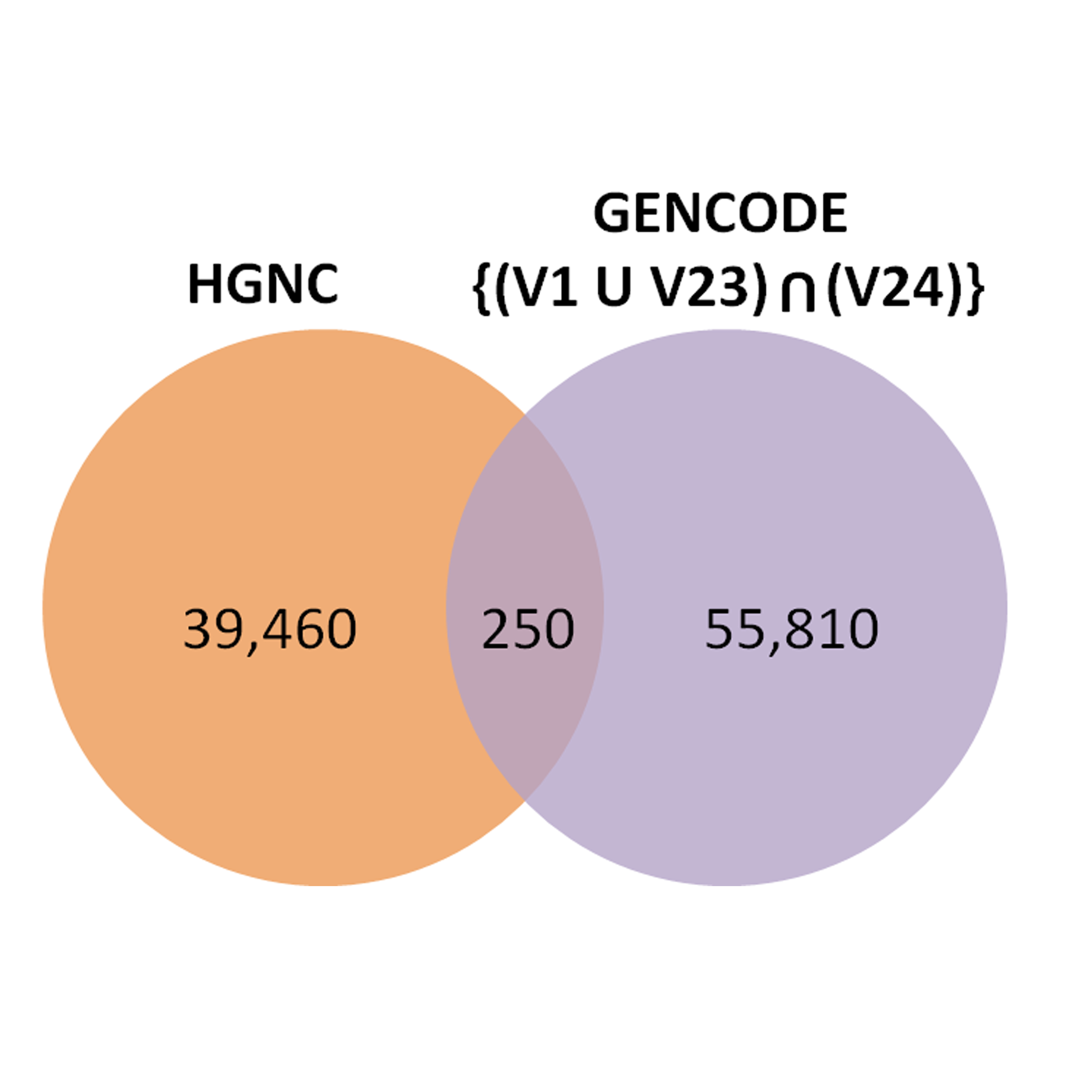

Supplement: Additional file 4: — Figure S4. Common and unique annotated genes of absent in GENCODE V24 and HGNC. Venn diagram shows intersection between genes annotated by GENCODE and HGNC. (JPG 1073 kb) [file 40246_2016_90_MOESM4_ESM.jpg]

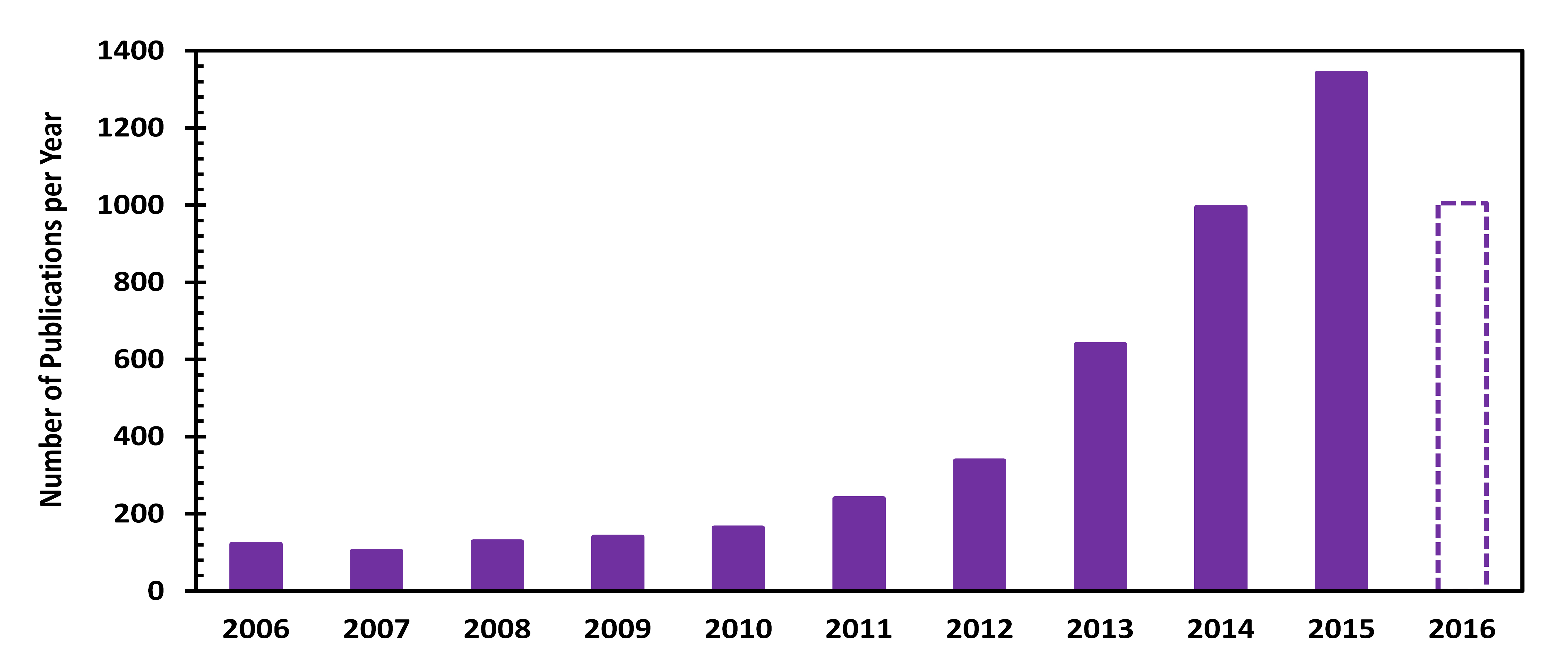

Supplement: Additional file 5: — Figure S5. Growth of literature in the field of lncRNAs. The number of publications for each year was retrieved using keyword “lncRNA” from PubMed. The data for 2016 is incomplete at the time of writing the manuscript and therefore marked with dotted lines. (JPG 1837 kb) [file 40246_2016_90_MOESM5_ESM.jpg]
